# Novel microbial synthesis of titania nanoparticles using probiotic *Bacillus coagulans* and its role in enhancing the microhardness of glass ionomer restorative materials

**DOI:** 10.1007/s10266-024-00921-5

**Published:** 2024-03-30

**Authors:** Afsheen Mansoor, Emaan Mansoor, Mazhar Mehmood, Syed Mujtaba Ul Hassan, Atta Ullah Shah, Uzma Asjid, Muhammad Ishtiaq, Asif Jamal, Akhilesh Rai, Paulo J. Palma

**Affiliations:** 1 Department of Microbiology, Quaid-I-Azam University, Islamabad, 45320 Pakistan; 2 Department of Dental Material Sciences, School of Dentistry, Shaheed Zulfiqar Ali Bhutto Medical University, Islamabad, 44080 Pakistan; 3 Islamic International Dental College, Riphah International University, Islamabad, 46000 Pakistan; 4 Department of Metallurgy and Materials Engineering, Pakistan Institute of Engineering and Applied Sciences, Islamabad, Pakistan; 5 Department of Materials, National Institute of Lasers and Optronics, Islamabad, 45650 Pakistan; 6 CNC-Center for Neuroscience and Cell Biology, CIBB-Center for Innovative Biomedicine and Biotechnology, University of Coimbra, 3004-504 Coimbra, Portugal; 7 Center for Innovation and Research in Oral Sciences (CIROS), Faculty of Medicine, University of Coimbra, 3000-075 Coimbra, Portugal; 8 Institute of Endodontics, Faculty of Medicine, University of Coimbra, 3000-075 Coimbra, Portugal

**Keywords:** *Bacillus coagulans*, Glass ionomer cements, Surface morphology, Titania NPs (TiO_2_), Vickers microhardness

## Abstract

Dental caries is a commonly occurring non-communicable disease throughout the world that might compromise the quality of any individual’s life. Glass ionomer cements (GIC) are the most acceptable restorative materials due to their ease of manipulation, minimal tooth loss and least invasive strategy; however, they lack mechanical stability that has become a point of concern. Nanoparticles (NPs) are an outstanding option for modifying and enhancing the properties of dental materials. The focus of this study was to prepare novel, biocompatible titania dioxide (TiO_2_) NPs as a dental-restorative material using an efficient probiotic *Bacillus coagulans*. The prepared NPs were incorporated into glass ionomer restorative material at varying concentrations and investigated for cell viability percentage, microhardness and surface morphology. Results indicated that pure 100% anatase phase TiO_2_ NPs with particle size of 21.84 nm arranged in smooth, spherical agglomerates and clusters forms. These NPs depicted cell viability > 90%, thus confirming their non-cytotoxic behavior. GIC restorative materials reinforced by 5% titania (TiO_2_) NPs demonstrated the highest microhardness in comparison to the control group and other experimental groups of the study. Surface morphology analysis revealed a reduction in cracks in this novel dental-restorative material supporting its compatible biological nature with better hardness strength and negligible crack propagation. Overall, these results indicated that TiO_2_ NPs produced using a biological approach could be easily used as restorative materials in dental applications.

**Graphical Abstract:**

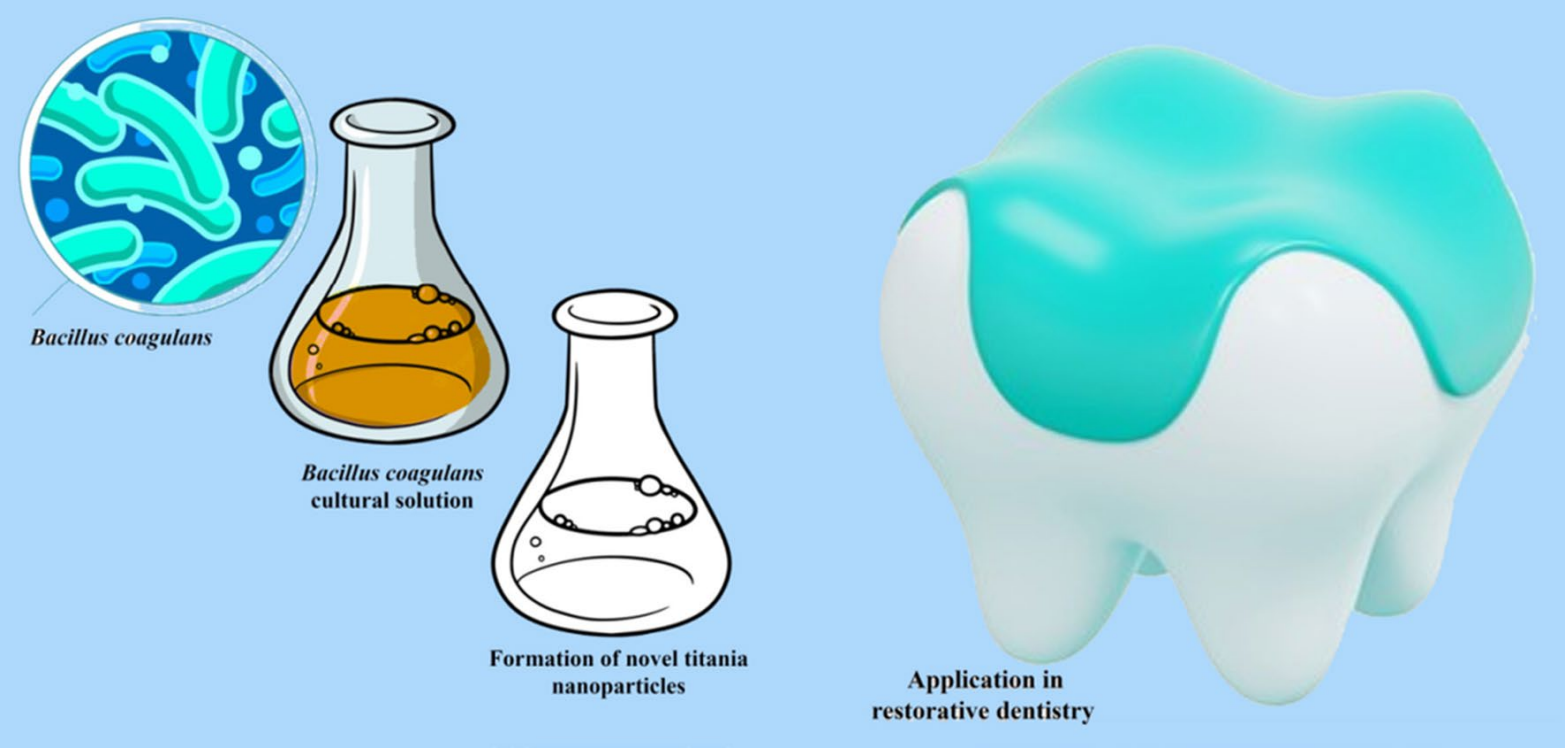

**Supplementary Information:**

The online version contains supplementary material available at 10.1007/s10266-024-00921-5.

## Introduction

Poor oral hygiene adversely affects the health of an individual and causes dental caries as reported by the World Health Organization (WHO) [1]. Invasive restorative treatments are frequently used to treat dental caries. Glass ionomer cements (GIC) have long been used for restoration purposes due to their good adherence potential to teeth. The binding ability of these materials to tooth structure enables them to be placed in cavities without creating any gaps, thus preventing the unnecessary tooth structure loss [2]. Moreover, these cements have the capability of releasing fluoride that helps in remineralization of teeth, consequently preventing secondary caries. Additionally, the color of these restorative materials is quite close to that of human teeth [3]. These materials display lower strength; therefore, efforts have been made to improve their mechanical properties in the past resulting in modifications of these cements such as resin-modified glass ionomer cements (RMGIC) and compomer [4]. These cements have higher mechanical strength as compared to conventional glass ionomer cements, but lack biocompatible behavior with adjacent biological tissues. The GIC is a potential bioactive dental material because of its higher favorable biocompatibility with oral tissues [5]. However, these materials need to be strengthened to act as long-term restorative materials due to their inferior mechanical qualities.

The composition of GIC may be responsible for its reduced strength. It is made of ion-leachable fluoroaluminosilicate glasses and polyacrylic acid solution that form a composite gel phase matrix eventually. An acid–base reaction forms this phase matrix, which does not bind all glass particles together and leaves some unreacted glass particles in the structure of set fluoroaluminosilicate glass and polyacrylic acid. These unreacted glass particles in the GIC matrix are responsible for the hydrolysis of the remaining tightly bound set glass structure [6]. This process disturbs bonding in the established glass structure of the GIC matrix, resulting in the release of calcium and aluminum ions. Cross-linking of the polymer chains in the GIC matrix is maintained by these ions, which are responsible for the ordered arrangement of the restorative material [6, 7]. The cross-linking in the GIC matrix is attributed to its microhardness properties. The loss of cross-linking in the GIC matrix by release of calcium and aluminum ions create voids in its structure and allows ingress of oral fluids and saliva dissolving its structure and compromising its mechanical properties [8]. Therefore, improving the mechanical properties of the GIC materials is one of the important aspects of future research and development. Nanotechnology-based approaches have significant contributions in modifying the quality of medical and dental materials. The incorporation of NPs in restorative materials and endodontic materials, mouthwashes, dentifrices, implants and luting cements has led to an enhanced performance of these materials [9–11].

Therefore, these NPs can be integrated in glass ionomer cements to improve their properties and strength, consequently improving their longevity. Various NPs such as silver (Ag), stainless steel, TiO_2_, tin and carbon have been used to enhance the properties of these restorative materials. Importantly, metallic NPs have gained exceptional success in dentistry due to their magnetic, mechanical, optical and strength properties [9–11]

TiO_2_ NPs have gained significant commercial attraction for various applications because of their chemical stability, high electrical conductivity, high thermal diffusivity and low thermal conductivity [12]. In dentistry, commercially available TiO_2_ NPs are considered as cost-effective, attrition controlling, fatigue resistant and corrosion-resistant materials for their application along with conventional restorative substances, but their purity is questionable as these NPs have displayed toxicity [8, 12]. Their added advantages and excellent mechanical properties make them ideal candidates for the formulation of improved dental-restorative materials. In recent years, incorporation of TiO_2_ NPs in dental restorations has immensely increased owing to their physical, chemical, mechanical and strength properties [13]. Many contemporary researchers have suggested that TiO_2_ NPs display potent anti-bacterial, anti-parasitic, anti-inflammatory, anti-fungal and anti-cariogenic properties [14]. TiO_2_ NPs are capable of producing desirable changes in the composition, structure, texture and topography of the GIC cement through strong bonding with its constituents. Therefore, modified GIC could attain various important properties, which are lacking in the absence of NPs [15]. One of the major limitations of the NPs could be the toxicity of the metals, which has been reported previously. Moreover, synthetic synthesis of these NPs has harmful effects on the environmental safety as a result of chemical by-product formation [16–18]. Therefore, an eco-friendly route for the fabrication of these NPs would be a step toward a safe and healthy environment. In this view of environmental biosafety, compatibility and stability, biological synthesis has gained ample interest in the current era. Therefore, *Bacillus coagulans* was used to produce TiO_2_ NPs for this study*. Bacillus coagulans* is an ideally effective probiotic that is easily available, highly safe and stable in nature. This microbially oriented synthesis would produce eco-friendly, pure, sustainable and environmentally safe NPs [19]. It displays strong antimicrobial activity against multiple pathogenic bacteria, as a result of its enhanced potency of releasing bacteriocin and acetic and lactic acid, which are taken as strong inhibitory compounds to kill microorganisms [20]. The current research focuses on the synthesis and characterization of novel TiO_2_ NPs via *Bacillus coagulans*, which were introduced into the conventional GIC cement to produce a novel compatible TiO_2_ GIC oral restorative material. The formulated GIC material was investigated for its microhardness and surface topography for its applications as a mechanically better and more durable treatment option.

## Materials and methods

### Synthesis

*Bacillus coagulans* (Accession No: ATCC^®^7050^™^, Catalog No: 0596P Micro biologics, Thermo Fisher Scientific, USA) was used in the synthesis of TiO_2_ NPs. The nutrient agar plates were freshly cultured with *Bacillus coagulans*. This culture was incubated at 37 °C for 24 h to obtain fresh strains of *Bacillus coagulans* that were mixed in 100 mL of nutrient broth in a flask and incubated at 28 °C with 150 rpm for 24 h to obtain a bacterial culture solution. Subsequently, 80 mL bacterial culture solution and 20 mL of 0.0025 M Ti(OH)_4_ solution (American Elements, 10,884-Weyburn Ave, Los Angeles, CA, USA) were mixed together to acquire a viscous mixture which was heated at 60 °C for 20 min. The white particles appeared at the bottom of the flask, revealing the synthesis of TiO_2_ NPs that were allowed to cool to room temperature for about 12–48 h [7].

### Characterization

Different equipment were employed for the characterization of TiO_2_ NPs to evaluate the physico-chemical properties, morphology and topology. They were investigated by X-ray diffraction analysis (DP-MAXZ 2400/Diffractometer, Rigaku Corporation, Akishima, Tokyo, Japan), scanning electron microscopy and energy-dispersive X-ray spectroscopy (Nova nanosem 430; Fei company 4022 261 49,391-S column F&G stron prep, Hillsboro, OR, USA), UV–Vis diffuse reflectance spectroscopy (Perkin Elmer, UV/Vis/NIR Spectrometer Lambda 950 Waltham, MA, USA), atomic force microscopy (Quesant Universal SPM, Ambios Technology, Santa Cruz, CA, USA), Fourier transmission infrared spectroscopy (JASCO FT/IR-6600, Ultrech-Amsterdam, AMS, Netherlands) and dynamic light scattering (Zeta sizer-nano Z-S Apparatus, ZEN-36000, Malvern panaLytical, Malvern-UK) [7, 8, 21]. The crystalline size and phase of the TiO_2_ NPs were evaluated by X-ray diffraction analysis and dynamic light scattering. Scanning electron microscopy and atomic force microscopy were utilized to analyze the surface morphology. Fourier transmission infrared spectroscopy demonstrated the purity of functional groups and compounds. The presence of elements in the composition of these NPs was confirmed via energy-dispersive X-ray spectroscopy. UV–VIS diffuse reflectance spectroscopy showed the particle size through band-gap energy value.

### Cytotoxicity evaluation

L929 mouse fibroblasts (ATCC; Manassass, V/A, USA) were kept in standard culture conditions to evaluate the cytotoxicity via the MTT assay (Sigma-Aldrich, Saint Louis, MO, USA). These fibroblasts were kept in 95% humidity and 37ºC temperature in 5.0% CO_2_ where 10% DMEM containing 1.0 × 10^4^ cells was used to make a cell suspension. Then, 100 µL cell suspension was seeded in every well of the standard 96-well plate for at least 24–48 h. Four different concentrations of TiO_2_ NPs (25–100 µg/mL) were prepared from 1.0 mg/mL stock solution of these TiO_2_ NPs to evaluate the cytotoxicity by adding an MTT assay into every well which was incubated at 37ºC for 2 h. Fluorescence well plate reader (Thermo Fisher’s, Waltham; MA, USA) was used to measure the fluorescence of every well at 490 nm wavelength after 24, 48 and 72 h [22, 23]. Different concentrations of TiO_2_ NPs were used as an experimental group and water was utilized as a control group. The cell viability rate of TiO_2_ NPs was calculated by:

$${\text{Cell viability }}\% \, = \,\frac{{{\text{mean optical density of the test group}}\, \times \,{1}00\% }}{{\text{mean optical density of the control group}}}$$

The calculated cell viability greater than 90.0% showed non-cytotoxicity, calculated cell viability between 60.0 and 90.0% revealed mild cytotoxicity, calculated cell viability between 30.0 and 60.0% depicted moderate cytotoxicity and calculated cell viability less than 30.0% confirmed severe cytotoxicity.

### Microhardness strength testing

#### Sample preparation

The commercial conventional GIC (GC Fuji Universal Gold Label 2) was used in this study whose composition is given in Table 1. A standard metal mold cylinder of about 9.5 × 1 mm was prepared for producing TiO_2_ GIC cylindrical samples (*n* = 50) to test the Vickers microhardness. Different concentrations of TiO_2_ NPs were added to GIC and mixed with liquid GIC to get TiO_2_ GIC cylinder samples, which were named conventional control group E-1 (0% TiO_2_ GIC), experimental group E-2 (3% TiO_2_ GIC), experimental group E-3 (5% TiO_2_ GIC), experimental group E-4 (7% TiO_2_ GIC) and experimental group E-5 (10% TiO_2_ GIC). The percentage distribution of commercial conventional GIC and TiO_2_ NPs is shown in Table 2. These 9.5 × 1 mm TiO_2_ GIC cylinders were then embedded in epoxy resin blocks and dried for about 24 h. These samples were then polished with silicone carbide papers of 400, 600 and 1000 grit [13].

**Table 1:** Composition of conventional GIC

Powder	Liquid
Alumina (Al_2_O_3_)	Polyacrylic acid
Silica oxide (SiO_2_)	Tartaric acid
Calcium fluoride (CaF_3_)	Water
Aluminum fluoride (AlF_3_)	
Sodium fluoride (NaF)	
Aluminum phosphate (AIPO_4_)

**Table 2:** Experimental groups containing conventional GIC and different percentages of TiO_2_ NPs

Group	Conventional GIC and percentage of TiO_2_ NPs
Conventional control group E-1 (0% TiO_2_ GIC)	5-g conventional GIC
Experimental group E-2 (3% TiO_2_ GIC)	4.85-g conventional GIC + 0.15-g TiO_2_ NPs
Experimental group E-3 (5% TiO_2_ GIC)	4.75-g conventional GIC + 0.25-g TiO_2_ NPs
Experimental group E-4 (7% TiO_2_ GIC)	4.65-g conventional GIC + 0.35-g TiO_2_ NPs
Experimental groupE-5 (10% TiO_2_ GIC)	4.50-g conventional GIC + 0.5-g TiO_2_ NPs

#### Vickers microhardness testing

The Vickers microhardness tester (Model: 401 Mud, S/N: 414, Wolpertw group, Atlanta, USA) was utilized as suggested by ISO 9917-1:2007 to obtain accurate measurements of microhardness for all samples of TiO_2_ GIC prepared cylinders as mentioned above. These TiO_2_ GIC prepared cylinders were placed one by one under a Vickers microhardness tester and a 3 N force was applied for 15 s to record their microhardness. Three readings were taken on one sample and then the average was estimated [13].

#### Scanning electron microscope (SEM) analysis

All samples such as conventional control group E-1, E-2, E-3, E-4 and E-5 were removed from epoxy resin blocks after carrying out Vickers microhardness testing, which were then finished, cleaned and sputter coated in a sputter coating machine (Quorum: Technologies; Ltd. Ashford, Ken; England) for at least 30 min. Then, all these samples were observed for cracks in SEM (Nova NanoSEM 430; Fei Company 4022 261 49391-S column F&G stron prep, Hillsboro, OR, USA) at different magnifications [13].

#### Statistical analysis

Statistical analysis in the current study was done by IBM SPSS v 24.00 (IBM Corporation, Armonk, NY, USA). One-way ANOVA and post hoc Tukey were performed for multiple comparisons where the significance was kept at *p* value < 0.05.

## Results

### Synthesis and characterization of TiO_2_ NPs

**Fig. 1:**
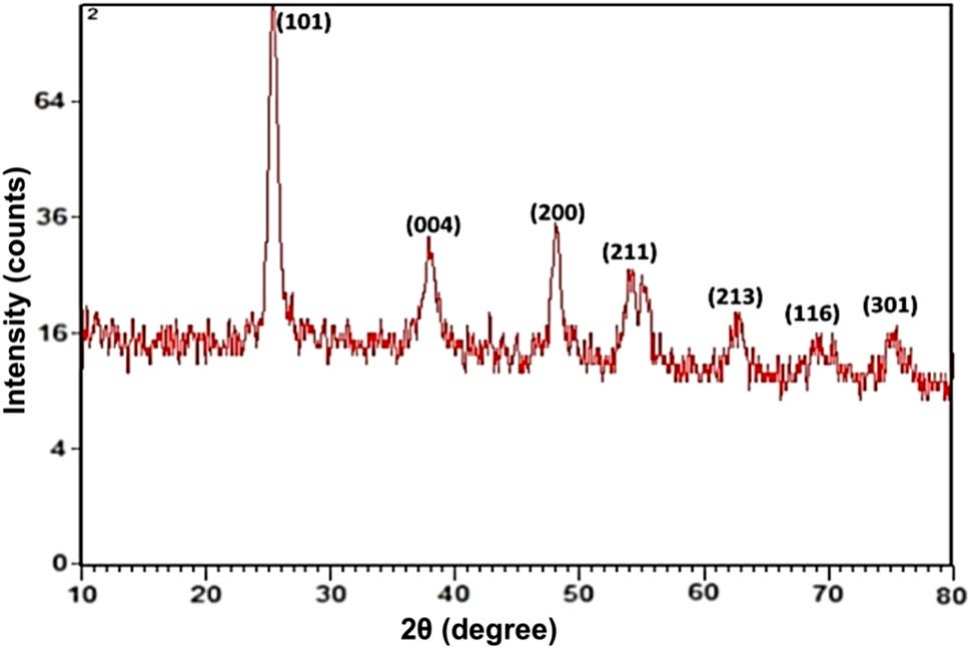
XRD pattern scan of TiO_2_ NPs synthesized by *Bacillus coagulans* showing different peaks

**Fig. 2:**
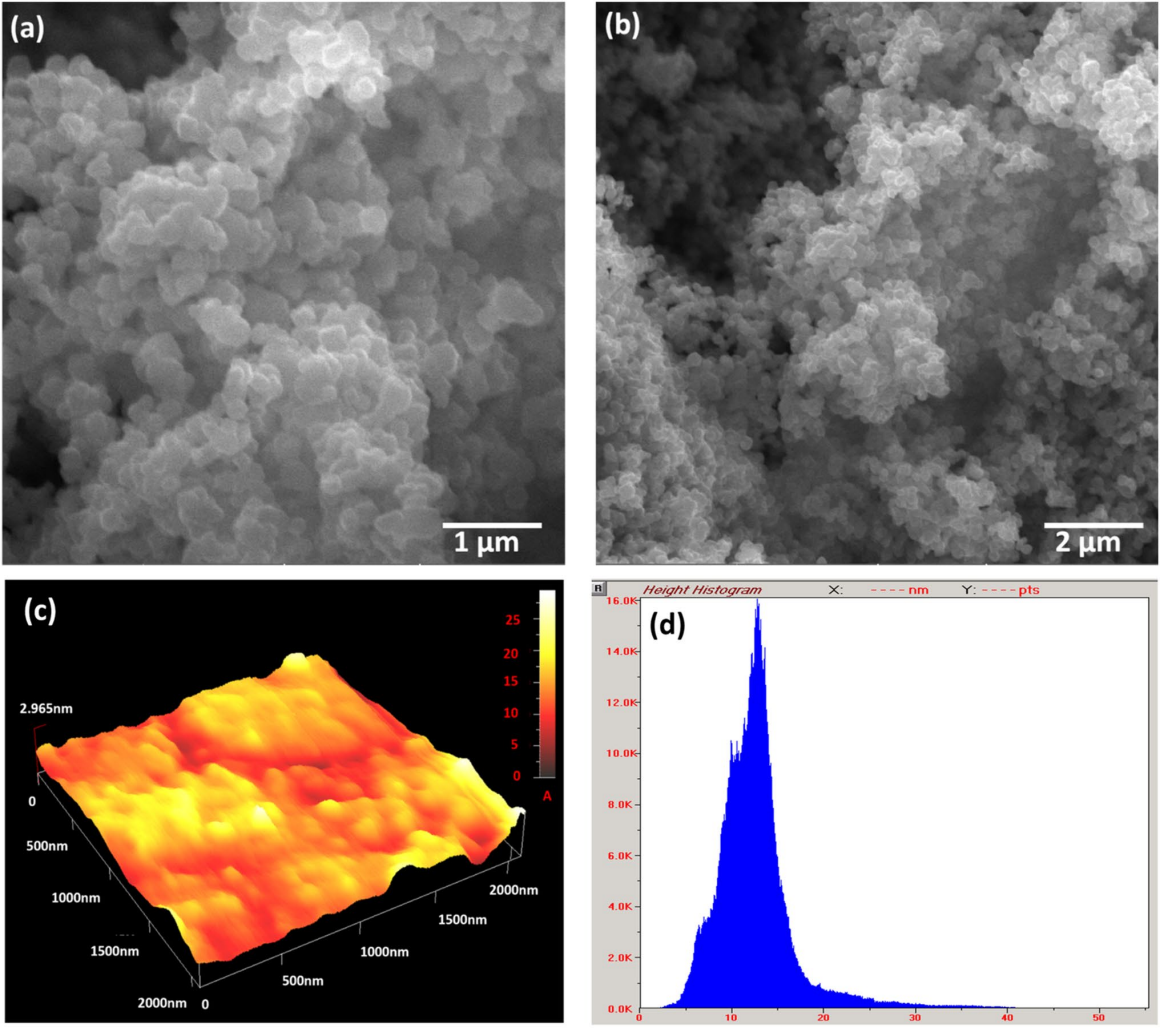
SEM image of TiO_2_ NPs synthesized by *Bacillus coagulans* at magnifications of **a** 50 kx, **b** 20 kx, **c, d** 3D AFM image and histogram of TiO_2_ NPs

The *Bacillus coagulans* culture solution was yellowish cream, which changed to white solution after the synthesis of TiO_2_ NPs (Supplementary Fig. 1). First, XRD was performed to characterize the crystalline phase of TiO_2_ NPs. Crystalline size and phase of TiO_2_ NPs were found to be in accordance with file no 01-071-1167 in its XRD pattern, which revealed a main peak (101) of the anatase phase at 25.38^◦^ (Fig. 1). Other peaks of TiO_2_ NPs belonging to the anatase phase were obtained at 004 = 37.98^°^, 200 = 48.14^°^, 211 = 54.12^°^, 213 = 62.77^°^, 116 = 68.59^°^ and 301 = 75.17. Thus, TiO_2_ NPs were in pure 100% anatase phase with the particle size of 21.84 nm (Fig. 1). SEM images show that the morphology of the prepared TiO_2_ NPs was predominantly spherically shaped particles in agglomeration states, which was well in accordance with AFM analysis (Fig. 2).

**Fig. 3:**
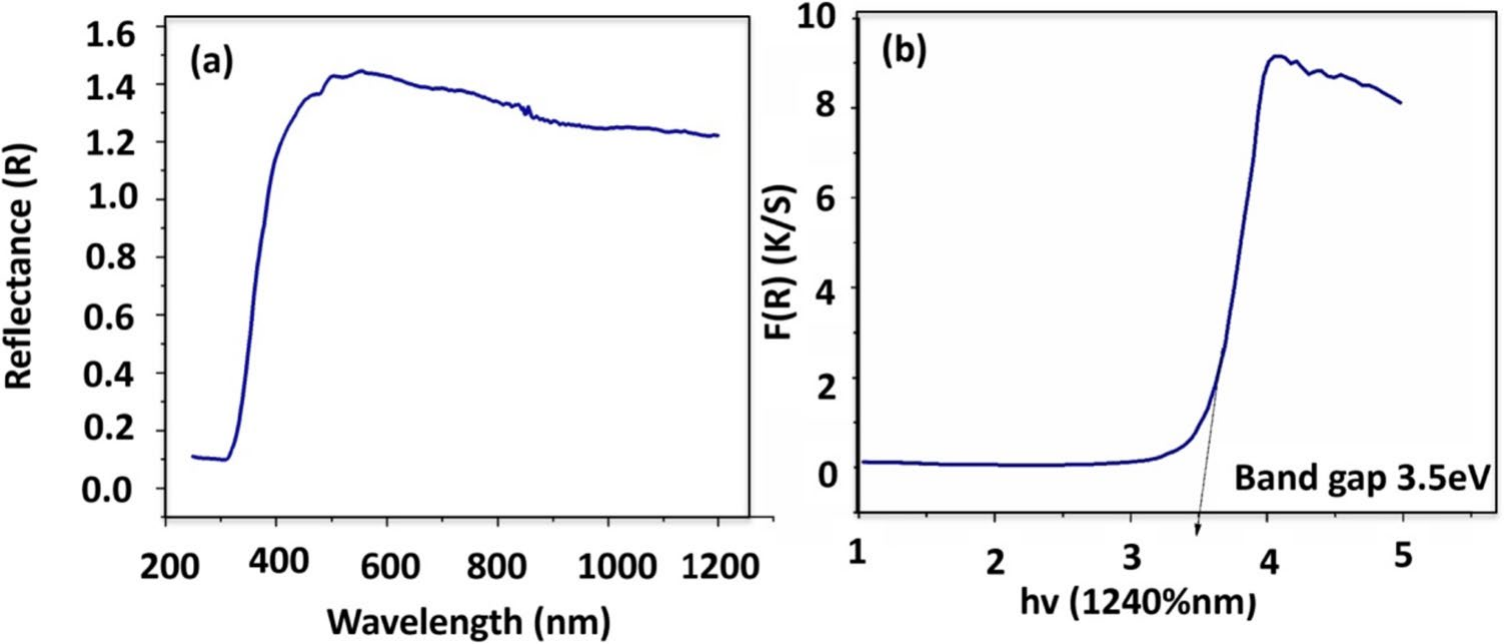
DRS pattern scan demonstrates **a** absorbance wavelength for formation and **b** energy band gap of TiO_2_ NPs

Additionally, DLS analysis revealed the hydrodynamic particle size of 34 nm of TiO_2_ NPs synthesized by *Bacillus coagulans* (Supplementary Fig. 3). UV/Vis DRS gives information about the crystal size of NPs through band-gap energy with standard value of 3.2 eV. The particle size and band gap value are in inverse relationship, which means that large band gap value contributes to small particle size of NPs and vice versa. The formation of TiO_2_ NPs prepared by *Bacillus coagulans* was at 320 nm and the band-gap energy of these NPs was 3.5 eV. This attributed toward the small crystallite size of these NPs (Fig. 3).

**Fig. 4:**
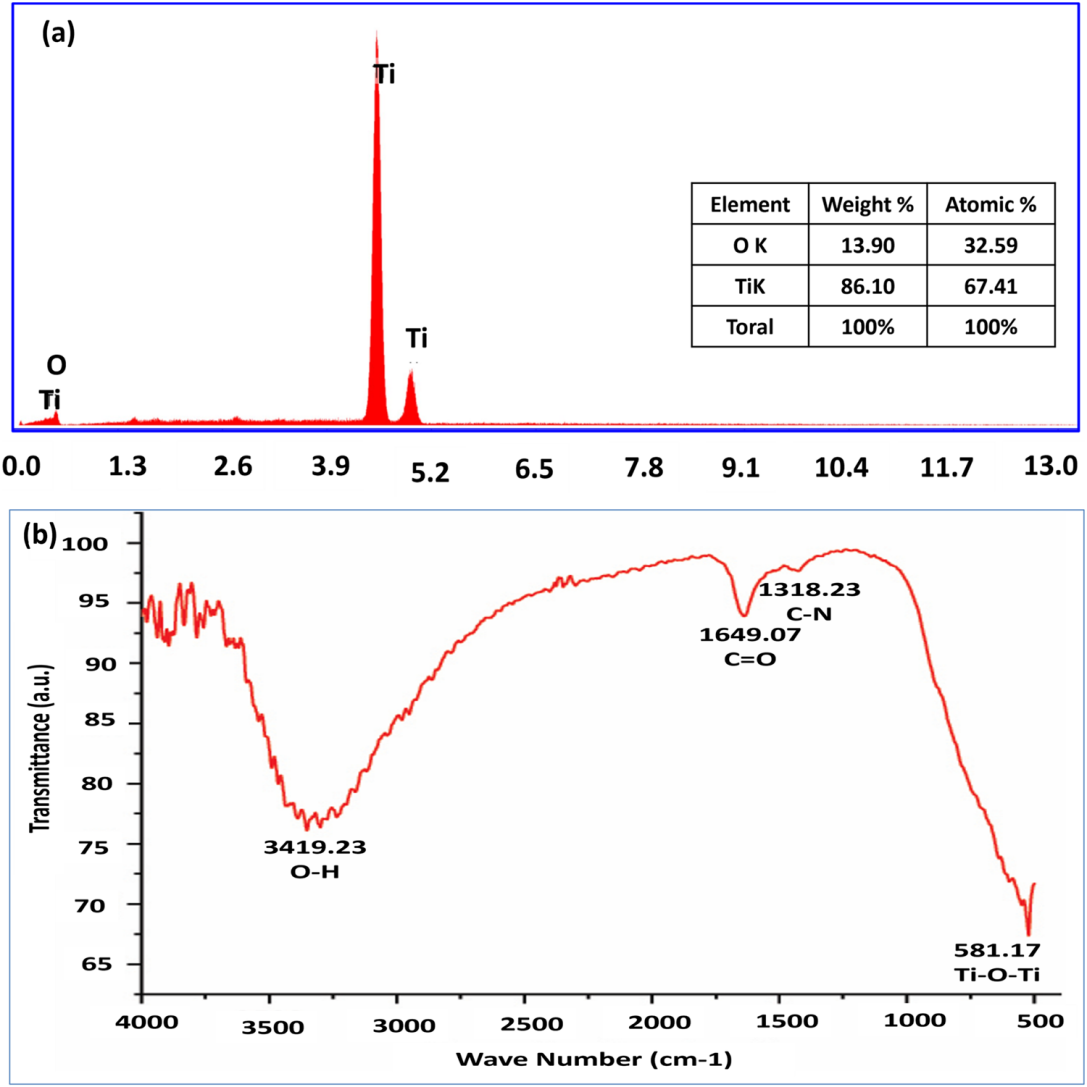
Spectrum scans of TiO_2_ NPs displaying its purity: **a** EDX scan revealing pure titanium and oxygen in its peak, **b** FTIR scan showing pure functional compounds and groups in is peaks

Elemental compositions in the form of intense peaks of Ti and O_2_ were observed in the EDX spectrum of TiO_2_ NPs synthesized by *Bacillus coagulans*. The quantity of Ti available was 86.10 weight% and 67.41 atomic%, while the quantity of O_2_ was 34.36 weight% and 50.64 atomic%, indicating the presence of TiO_2_ NPs (Fig. 4a). Functional compound analysis demonstrated the major notable peaks in the FTIR spectrum of the prepared TiO_2_ NPs at 3419.23 cm^−1^_,_ 2791.09 cm^−1^_,_ 1649.07 cm^−1^ and 581.17 cm^−1^. The peak 3419.23 cm^−1^ represents the O–H stretching vibrations of alcohol groups, while the peak 2791.09 cm^−1^ reveals C–H bending of an aromatic compound. The peak at 1649.07 cm^−1^ corresponds to the group of amines and the peak 581.17 cm^−1^ is attributed to Ti–O–Ti bending, ensuring the formation of TiO_2_ NPs. These additional functional groups and compounds observed in the FTIR spectrum of TiO_2_ NPs confirmed their purity (Fig. 4b).

### Cytotoxicity evaluation

**Fig. 5:**
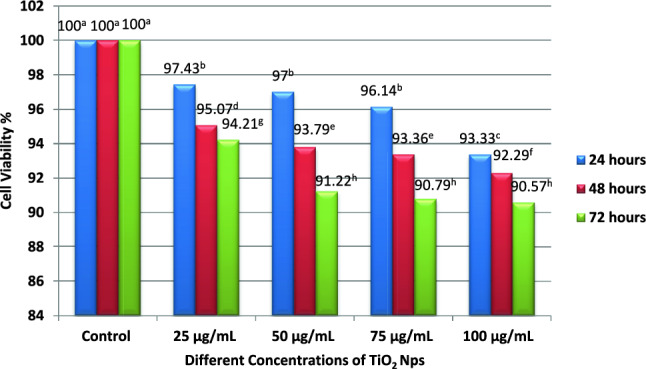
Different concentrations of TiO_2_ NPs revealing differences in cell viability percentage after 24, 48 and 72 h of cytotoxicity evaluation, where *p* value > 0.05 for same alphabets in superscripts and *p* value < 0.05 for different alphabets in superscripts

Cell viability of four different concentrations of TiO_2_ NPs, i.e., 25 µg/mL, 50 µg/ml, 75 µg/mL and 100 µg/mL, was investigated after 24, 48 and 72 h. The control group revealed 100% cell viability on all the investigated days. The 25 µg/mL concentration of TiO_2_ NPs displayed cell viability percentage of 97.43%, 95.07% and 94.21%, whereas 50 µg/mL concentration of TiO_2_ NPs revealed cell viability percentage of 97%, 93.79% and 91.22% after 24, 48 and 72 h. Moreover, 75 µg/mL concentration of TiO_2_ NPs displayed cell viability percentage of 96.14%, 93.36% and 90.79%, whereas 100 µg/mL concentration of TiO_2_ NPs revealed cell viability percentage of 93.33%, 92.29% and 90.57% after 24, 48 and 72 h. The inter-group difference in the cell viability percentage among the 25 µg/ml, 50 µg/mL, and 75 µg/mL concentrations of TiO_2_ NPs was insignificant (*p* > 0.05) but their cell viability percentage was found to be significant (*p* < 0.05) with 100 µg/mL concentration of TiO_2_ NPs after 24 h. In addition, the inter-group difference in the cell viability percentage among 25 µg/ml and 100 µg/mL concentration of TiO_2_ NPs was significant (*p* < 0.05), but it was calculated to be insignificant (*p* > 0.05) between the concentration of 50 µg/mL and 75 µg/mL TiO_2_ NPs after 48 h. On the other hand, the inter-group difference in the cell viability percentage among 25 µg/mL and other concentrations of TiO_2_ NPs was significant (*p* < 0.05), but it demonstrated insignificant (*p* > 0.05) results among the concentrations of 50 µg/mL, 75 µg/mL and 100 µg/mL TiO_2_ NPs after 72 h (Fig. 5).

### Microhardness strength testing of novel TiO_2_ GIC

#### Vickers microhardness analysis

**Fig. 6:**
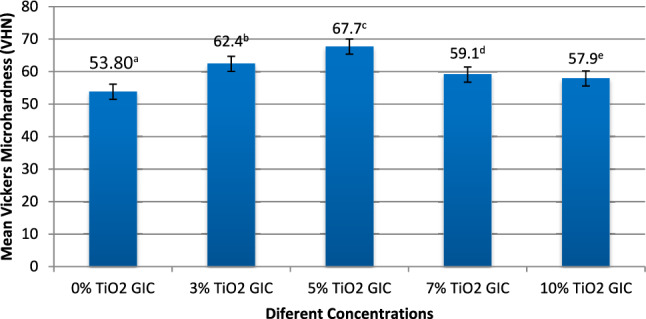
Differences in mean Vickers microhardness values of conventional control group E-1 (0% TiO_2_ GIC) and different concentrations of novel experimental group E-2 (3% TiO_2_ GIC), experimental group E-3 (5% TiO_2_ GIC), experimental group E-4 (7% TiO_2_ GIC) and experimental group E-5 (10% TiO_2_ GIC) with standard error (S.E) with *p* value < 0.05 for different alphabets in superscript form

The Vickers microhardness strength of conventional control group E-1 (0% TiO_2_ GIC), experimental group E-2 (3% TiO_2_ GIC), experimental group E-3 (5% TiO_2_ GIC), experimental group E-4 (7% TiO_2_ GIC) and experimental group E-5 (10% TiO_2_ GIC) was evaluated. Ten samples from each group were tested for the hardness strength. The Vickers microhardness strength revealed a linear increase with increasing % of TiO_2_ NPs in novel glass ionomer cement samples. This increase in the Vickers microhardness strength was confirmed till 5% TiO_2_ NPs addition in the experimental group E-3 (5% TiO_2_ GIC), which then revealed linear pattern declination in the Vickers microhardness strength with further increase in TiO_2_ NPs concentration in the experimental group E-4 (7% TiO_2_ GIC) and experimental group E-5 (10% TiO_2_ GIC). The conventional group E-1 displayed minimum mean Vickers microhardness value of about 53.80 ± 1.26 (S.E = 0.39) VHN, whereas other experimental group E-2, E-3, E-4 and E-5 samples displayed mean Vickers microhardness values of about 62.40 ± 1.06 (S.E = 0.33) VHN, 67.70 ± 0.46 (S.E = 0.15) VHN, 59.10 ± 0.87 (S.E = 0.27) VHN and 57.90 ± 1.08 (S.E = 0.34) VHN, respectively. Thus, the novel experimental group E-3 exhibited maximum microhardness in comparison to the conventional control group E-1 and other experimental groups E-2, E-4 and E-5 samples, which was significant (*p* < 0.05) (Fig. 6).

The mean differences between Vickers microhardness of conventional group E-1 and TiO_2_ NPs-based groups such as E-2, E-3, E-4 and E-5 were significant. The inter-group comparisons were statistically significant between E-1, E-2 and E-3 TiO_2_-based groups (*p* = 0.001) and were also statistically significant between E-4 and E-5 TiO_2_-based groups (*p* = 0.009). The novel TiO_2_-based group E-3 restorative material had maximum increase in its microhardness (*p* = 0.001) (Table 3).

**Table 3:** Inter-group comparisons of Vickers microhardness displaying microhardness strength of different groups

Different concentrations of TiO_2_ GIC samples	Comparison of Vickers microhardness between different TiO_2_ GIC samples	Mean difference of Vickers microhardness with standard error (SE)	*P* value
Conventional control group E-1 (0% TiO_2_ GIC)	Vickers microhardness of the experimental group E-2 (3% TiO_2_ GIC)	−8.60 (0.44)	0.001
	Vickers microhardness of the experimental groupE-3 (5% TiO_2_ GIC)	−13.90 (0.44)	0.001
	Vickers microhardness of the experimental group E-4 (7% TiO_2_ GIC)	−5.30 (0.44)	0.001
	Vickers microhardness of the experimental group E-5 (10% TiO_2_ GIC)	−4.10 (0.44)	0.001
Experimental group E-2 (3% TiO_2_ GIC)	Vickers microhardness of the conventional control group E-1 (0% TiO_2_ GIC)	8.60 (0.44)	0.001
	Vickers microhardness of the experimental group E-3 (5% TiO_2_ GIC)	−5.30 (0.44)	0.001
	Vickers microhardness of the experimental group E-4 (7% TiO_2_ GIC)	3.30 (0.44)	0.001
	Vickers microhardness of the experimental group E-5 (10% TiO_2_ GIC)	4.50 (0.44)	0.001
Experimental group E-3 (5% TiO_2_ GIC)	Vickers microhardness of the conventional control group E-1 (0% TiO_2_ GIC)	13.90 (0.44)	0.001
	Vickers microhardness of the experimental group E-2 (3% TiO_2_ GIC)	5.30 (0.44)	0.001
	Vickers microhardness of the experimental group E-4 (7% TiO_2_ GIC)	8.60 (0.44)	0.001
	Vickers microhardness of the experimental group E-5 (10% TiO_2_ GIC)	9.80 (0.44)	0.001
Experimental group E-4 (7% TiO_2_ GIC)	Vickers microhardness of the conventional control group E-1 (0% TiO_2_ GIC)	5.30 (0.44)	0.001
	Vickers microhardness of the experimental group E-2 (3% TiO_2_ GIC)	−3.30 (0.44)	0.001
	Vickers microhardness of the experimental group E-3 (5% TiO_2_ GIC)	−8.60(0.44)	0.001
	Vickers microhardness of the experimental group E-5 (10% TiO_2_ GIC)	1.20 (0.44)	0.009
Experimental group E-5 (10% TiO_2_ GIC)	Vickers microhardness of the conventional control group E-1 (0% TiO_2_ GIC)	4.10 (0.44)	0.001
	Vickers microhardness of the experimental group E-2 (3% TiO_2_ GIC)	−4.50 (0.44)	0.001
	Vickers microhardness of experimental group E-3 (5% TiO_2_ GIC)	−9.80 (0.44)	0.001
	Vickers microhardness of the experimental group E-4 (7% TiO_2_ GIC)	−1.20 (0.44)	0.009

#### Surface morphology analysis

**Fig. 7:**
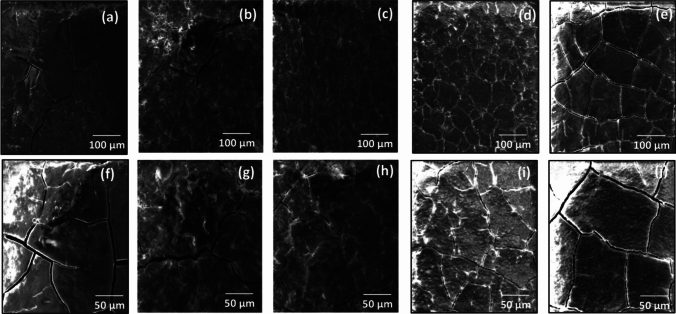
SEM images of TiO_2_ GIC samples E-1 (**a, f**), E-2 (**b, g**), E-3 (**c, h**), E-4 (**d, i**) and E-5 (**e, j**)

SEM analysis of all samples such as conventional control group E-1, E-2, E-3, E-4 and E-5 was carried out after Vickers microhardness testing for surface morphological changes such as cracks. The conventional control group E-1 revealed maximum crack sites (Fig. 7 a, f) due to reduced microhardness in the absence of TiO_2_ NPs. The experimental group E-2 (Fig. 7 b, g) revealed a slight deduction in crack sites. The deduction in cracks became maximum in the experimental group E-3 (Fig. 7 c, h) that became novel in this study as a result of its maximum microhardness. Further, an increase in the percentages of TiO_2_ NPs greatly reduced the microhardness and revealed increased crack sites in the experimental group E-4 (Fig. 7 d, i). The experimental group E-5 showed maximum crack sites because of minimum microhardness attained in this group (Fig. 7 e, j).

## Discussion

TiO_2_ NPs used in this study were fabricated by a novel microbial route using *Bacillus coagulans*. These TiO_2_ NPs were added to conventional dental glass ionomer cements to produce a novel compatible dental cement (5% TiO_2_ GIC) with increased microhardness. TiO_2_ NPs produced via microbial synthesis were considered biocompatible, sustainable, and environmentally friendly, as no toxic chemicals and artificial ingredients were involved in their synthesis. They were prepared at very low pressure and temperature. The production of TiO_2_ NPs is cost-effective, and thus they can be easily incorporated in the commercial restorative products and medicines used in dental and medical fields [24]. The oxidation reaction between the microbial culture solution and Ti(OH)_2_ was mainly responsible for the production of TiO_2_ NPs. The X-ray diffraction pattern revealed the TiO_2_ NPs were 100% pure anatase phase with a particle size of 21.84 nm. The 100% pure anatase phase of TiO_2_ NPs was produced due to their calcination at 500 °C in the furnace, resulting in their active and reactive phase. SEM analysis revealed spherical shape in agglomerates and clustered form of TiO_2_ NPs, while DLS measurement indicated the hydrodynamic diameter of 34 nm.

The formation of TiO_2_ NPs was observed at 320 nm, which was in accordance with the previous study, where formation of TiO_2_ NPs was confirmed between 200 and 600 nm [25]. The standard band-gap energy value (standard value = 3.2 eV) also confirms the formation of TiO_2_ NPs. Values higher than 3.2 eV demonstrate a smaller particle size of NPs, while values lesser than the recommended value demonstrated a larger particle size of NPs. The band-gap energy of 3.5 eV NPs could be due to the slow secondary reaction by the biomolecules (capping and reducing agents).

EDX analysis demonstrated prominent peaks of Ti and O with no other impurity in its composition due to the presence of *Bacillus coagulans* culture solution and Ti precursors [26]. FTIR analysis demonstrated the evident peak of Ti–O–Ti bending at 581.17 cm^-1^ ensuring the presence of TiO_2_ NPs [27]. The absence of C–H stretching peak, particularly at 2900 cm^−1^, confirmed the absence of any organic compound responsible for producing impurities that might lead to cytotoxicity. The beneficial effect of pure amine linkages might have occurred as a result of involvement of the proteins present in TiO_2_ NPs [28]. These novel TiO_2_ NPs prepared from microbial route revealed cell viability percentage > 90%, thus confirming their toxicity-free biocompatible nature. Previous studies reported the nontoxic behavior of the TiO_2_ NPs when synthesized from microbes [29, 30]. The strong biomolecular linkages during the fabrication might have generated a dominant overlapping aggregation process that prompted the stable nucleation and binding of the NPs, thus attributing to their characteristically safe, sustainable and impurity-free nature. The production of impurity-free TiO_2_ encourages their usage in restorative materials applied adjacent to the oral biological tissues.

The hardness of a dental material is a significantly essential property that may be used to validate its strength and longevity in the oral cavity [31, 32]. The current study showed maximum microhardness in the novel experimental group E-3 as compared to the conventional control group E-1, E-4 and E-5 (Table 3). This could be possible due to the fact that TiO_2_ NPs occupied the voids available in the glass ionomer matrix. Eventually, this could have resulted in strong bonding and cross-linking between pure and small-sized 5% TiO_2_ NPs and dental glass ionomer powder particles. The strong binding prevented propagation of cracks through the GIC when a force is applied to these NPs. The increase in microhardness of the E-3 group did not correspond to that in the literature [16] due to the high incorporation of TiO_2_ NPs. These NPs created a powerful meshwork to fill each void in the glass ionomer matrix by strongly entangling the matrix in an orderly arranged manner to prevent the loss of cross-linking and bond around the void.

The other experimental groups E-4 and E-5 revealed reduction in the microhardness, which were not evaluated previously [13] The reason behind the declination of microhardness could be the weak attractive forces between 7% TiO_2_ NPs and the GIC matrix in the E-4 and E-5 groups. The SEM analysis revealed a minor crack formation in the surface of group E-3, because of its maximum microhardness as compared to conventional control group E-1 that depicted major crack formation with minimum microhardness. This clearly indicated that any alteration in the composition of conventional dental GIC by smart NPs might have a significant effect on its microhardness and surface topographical structure. The amount of NPs added, particle size, distribution, cross-linking, chemical reaction and chemical bonding between TiO_2_ NPs and dental glass ionomer matrix played a potent role in enhancing the microhardness and surface topographical structure of group E-3.

## Conclusions

TiO_2_ NPs were prepared using probiotic *Bacillus coagulans* in an environmentally friendly manner. TiO_2_ NPs possessed 100% pure anatase phase with the particle size ranging between 21 and 34 nm. NPs were spherical in shape and arranged in agglomerates and clusters showing their smooth texture. TiO_2_ NPs were potently stable, sustainable and reproducible enough to enhance the microhardness strength of 5% TiO_2_ GIC dental glass ionomer cement. TiO_2_ incorporated cement displayed strong surface topographical structure with minimal pores that ensured its compatible biological nature with better hardness strength and negligible crack propagation.

## Supplementary Information

Below is the link to the electronic supplementary material.

Supplementary file1 (DOCX 3828 KB)

## Data Availability

All the data has been already shared in the manuscript.
