# Supplementary material for: Novel microbial synthesis of titania nanoparticles using probiotic Bacillus coagulans and its role in enhancing the microhardness of glass ionomer restorative materials
Source: Odontology. 2024 Mar 30;112(4):1123–34. doi: 10.1007/s10266-024-00921-5 (PMC11415460; doi:10.1007/s10266-024-00921-5)
Supplement: Supplementary file 1 — Supplementary file1 (DOCX 3828 KB) [file 10266_2024_921_MOESM1_ESM.docx]

**Supplementary information**

Novel microbial synthesis of titania nanoparticles using probiotic *Bacillus coagulans* and its role in enhancing microhardness of glass ionomer restorative materials

**
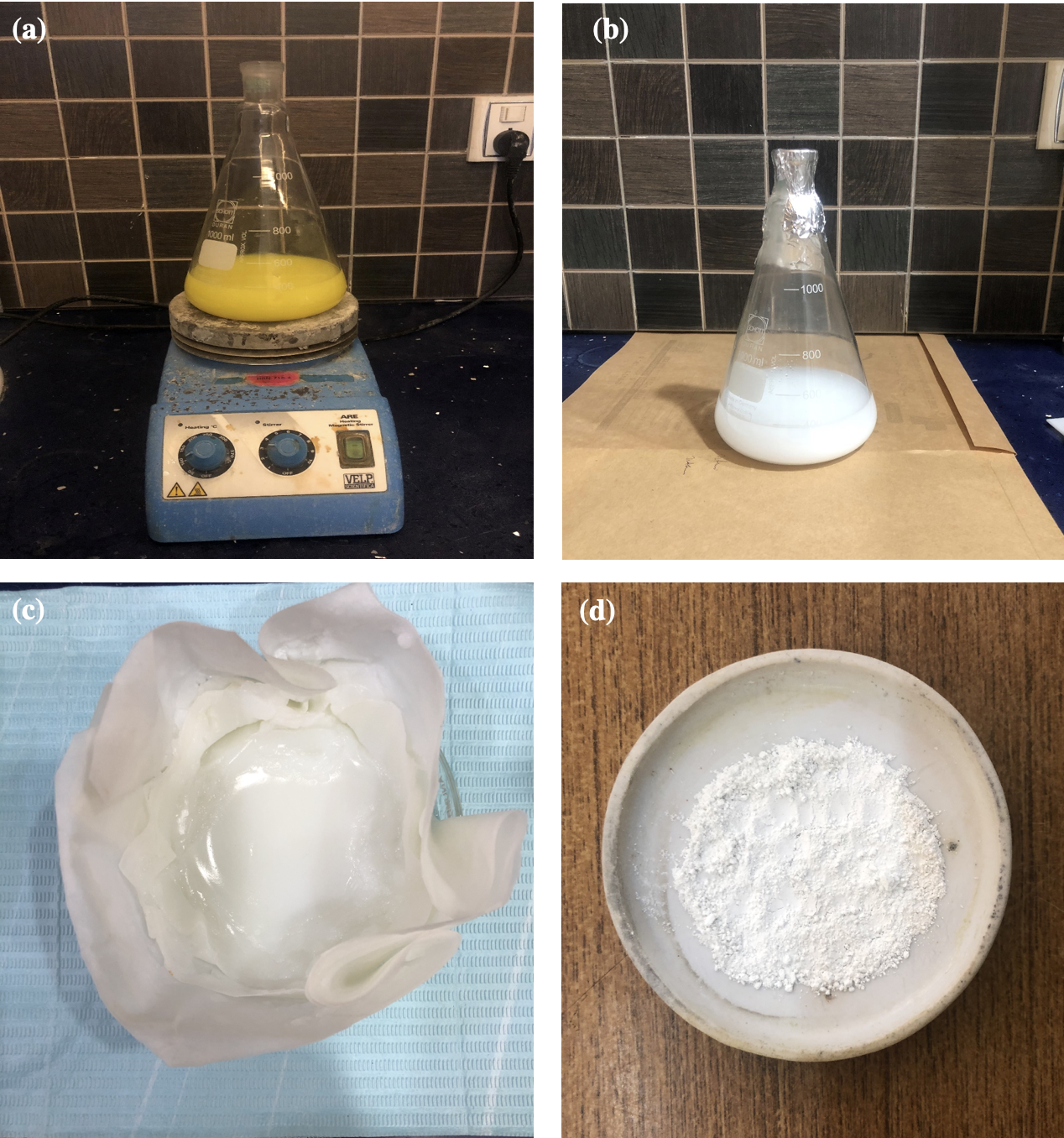
**

**Supplementary Figure 1:** Synthesis of TiO_2_ NPs: (a) *Bacillus coagulans* culture solution, (b) TiO_2_ NPs precipitates in flask, (c) cake form of TiO_2_ NPs and (d) powder form of TiO_2_ NPs.


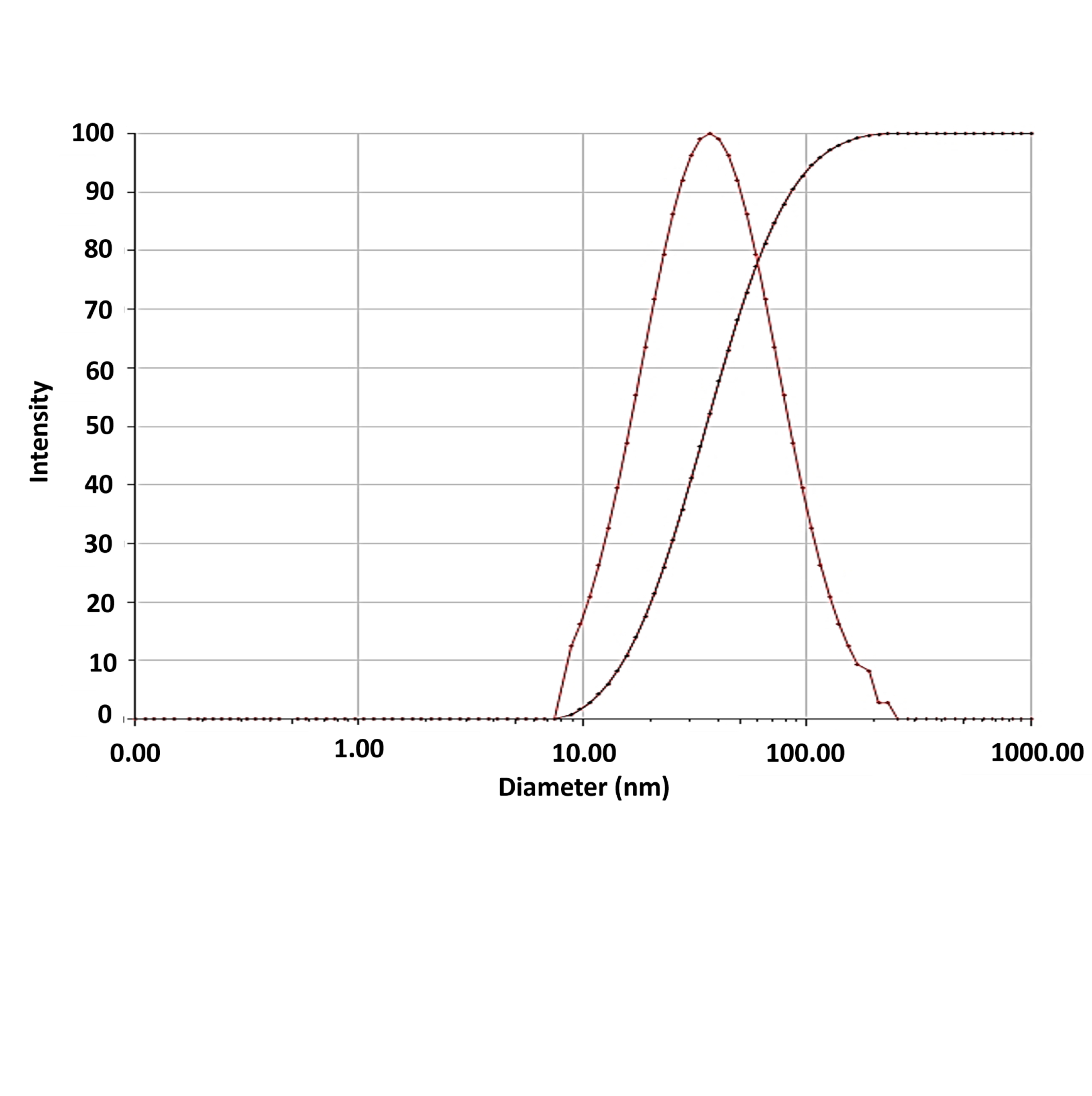


**Supplementary Figure 2:** DLS analysis of TiO_2_ NPs synthesized by *Bacillus coagulans*.


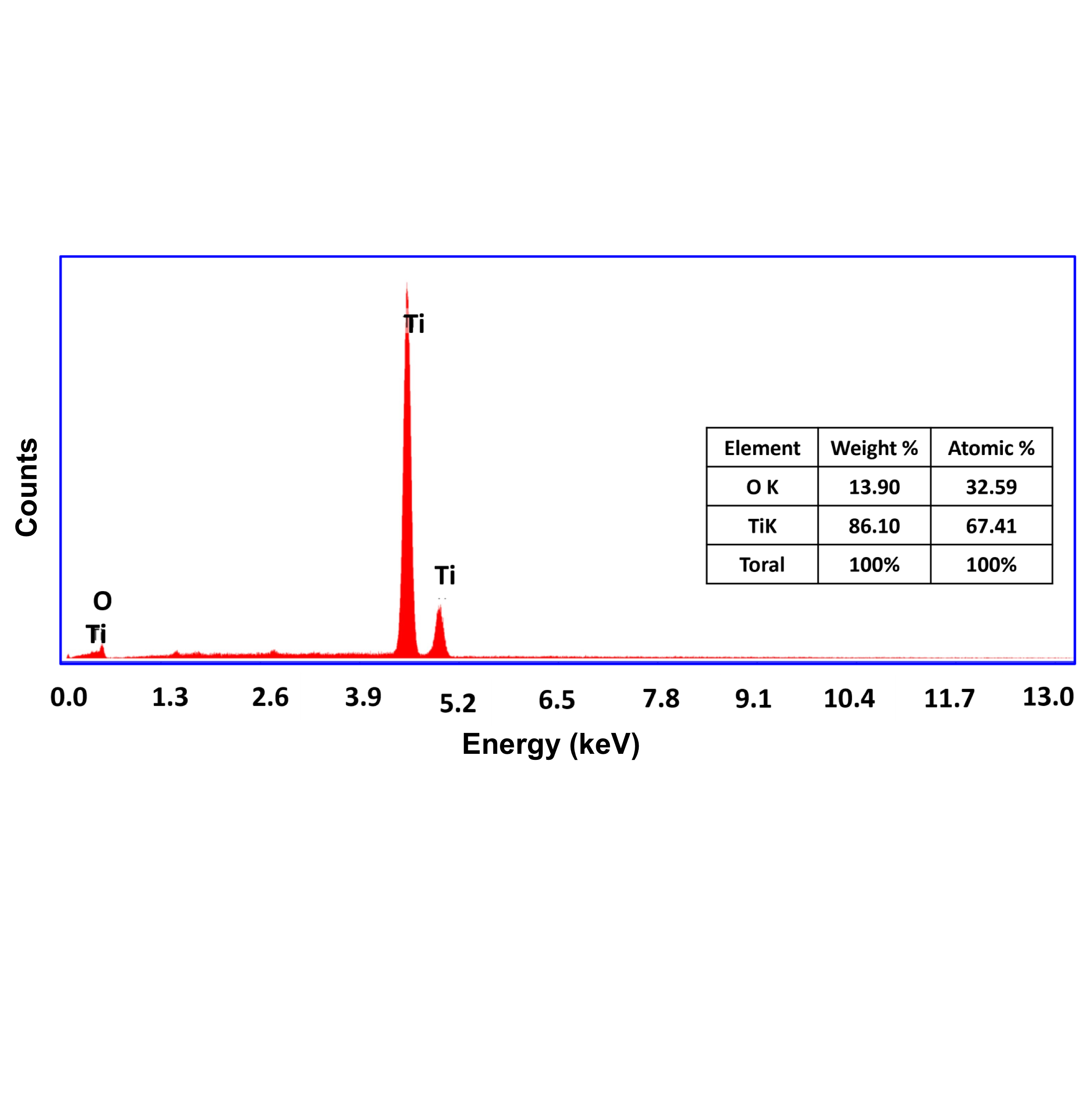


**Supplementary Figure 3:** Energy dispersive x-ray (EDX) analysis of TiO_2_ NPs synthesized by *Bacillus coagulans.*
